# GC-MS Based Characterization, Antibacterial, Antifungal and Anti-Oncogenic Activity of Ethyl Acetate Extract of *Aspergillus niger* Strain AK-6 Isolated from Rhizospheric Soil

**DOI:** 10.3390/cimb45050241

**Published:** 2023-04-24

**Authors:** Shaik Kalimulla Niazi, Dhanyakumara Shivapoojar Basavarajappa, Sushma Hatti Kumaraswamy, Asmatanzeem Bepari, Halaswamy Hiremath, Shashiraj Kariyellappa Nagaraja, Muthuraj Rudrappa, Anil Hugar, Mary Anne Wong Cordero, Sreenivasa Nayaka

**Affiliations:** 1 Department of Preparatory Health Sciences, Riyadh Elm University, Riyadh 12611, Saudi Arabia; kalimullaniazi@gmail.com; 2 Department of Studies in Botany, Karnatak University, Dharwad 580003, Karnataka, India; dhanyakumarsb@gmail.com (D.S.B.); hmhalaswamy2063@gmail.com (H.H.); rajscbz@gmail.com (S.K.N.); rmuthuraj20@gmail.com (M.R.); anilh5157@gmail.com (A.H.); 3 Department of Pharmacology, Jagadguru Jayadeva Murugarajendra Medical College (JJMMC), Davanagere 577004, Karnataka, India; drhksushma1987@gmail.com; 4 Department of Basic Health Sciences, College of Medicine, Princess Nourah bint Abdulrahman University, Riyadh 11671, Saudi Arabia; ambepari@pnu.edu.sa (A.B.); macordero@pnu.edu.sa (M.A.W.C.)

**Keywords:** rhizospheric soil, *Aspergillus niger*, antimicrobial, antifungal, GC-MS analysis, apoptosis

## Abstract

Rhizospheric soil is the richest niche of different microbes that produce biologically active metabolites. The current study investigated the antimicrobial, antifungal and anticancer activities of ethyl acetate extract of the potent rhizospheric fungus *Aspergillus niger* AK6 (AK-6). A total of six fungal isolates were isolated, and isolate AK-6 was selected based on primary screening. Further, it exhibited moderate antimicrobial activity against pathogens such as *Klebsiella pneumonia*, *Candida albicans*, *Escherichia coli*, *Shigella flexneri*, *Bacillus subtilis* and *Staphylococcus aureus*. The morphological and molecular characterization (18S rRNA) confirmed that the isolate AK-6 belonged to Aspergillus niger. Further, AK-6 showed potent antifungal activity with 47.2%, 59.4% and 64.1% of inhibition against *Sclerotium rolfsii*, *Cercospora canescens* and *Fusarium sambucinum* phytopathogens. FT-IR analysis displayed different biological functional groups. Consequently, the GC-MS analysis displayed bioactive compounds, namely, n-didehydrohexacarboxyl-2,4,5-trimethylpiperazine (23.82%), dibutyl phthalate (14.65%), e-5-heptadecanol (8.98%), and 2,4-ditert-butylphenol (8.60%), among the total of 15 compounds isolated. Further, the anticancer activity of AK-6 was exhibited against the MCF-7 cell line of human breast adenocarcinoma with an IC_50_ value of 102.01 μg/mL. Furthermore, flow cytometry depicted 17.3%, 26.43%, and 3.16% of early and late apoptosis and necrosis in the AK-6 extarct treated MCF-7 cell line, respectively. The results of the present analysis suggest that the isolated *Aspergillus niger* strain AK-6 extract has the potential to be explored as a promising antimicrobial, antifungal and anticancer drug for medical and agricultural applications.

## 1. Introduction

Most of the world’s population depends on natural products found in species of living macro-organisms, animals, marine species, micro-organisms and plants for primary healthcare and medicine. Although these bioactive molecules have perfect chemical diversity, less than 10% of the globe’s biodiversity has been evaluated for potential biological activity. Numerous beneficial natural lead compounds await discovery, with the challenge being accessing this natural chemical diversity [1].

Fungi, eukaryotic micro- and macro-organisms belonging to the Kingdom Mycota, have existed as an element of human life for thousands of years. Ancient people used fungi for various applications such as food, alcoholic beverages, medicine, and cultural purposes [2]. This fungal diversity is estimated to be 2.2 to 3 million; among these, there are 120,000 accepted species. In addition, numerous novel fungal species are being discovered in various habitats, such as the aquatic environment, tropical forest plants and soil associated with insects [3,4]. Soil is a bio-geochemically dynamic natural resource composed of inorganic and organic matter. The inorganic soil is comprised of rock broken down into small particles of sand, silt, and clay. In contrast, organic material constitutes organic compounds and contains living and dead plant, animal and microbial material. Standard soil has a biomass configuration of 70% micro-organisms, 22% macrofauna and 8% roots [5]. Soil micro-organisms are most commonly found in the rhizospheric region of the soil, an area around the root where many chemical and biochemical process occurs, and is a good source of soil for isolating fungus [6]. Fungi are successful soil inhabitants and form 500 to 5000 kg per hectare of biomass of all soil organisms, which is enormous [4], due to their high plasticity and ability to adopt diverse forms in reaction to unfavorable conditions [7]. Some common fungi in the soil are *Amantia*, *Aspergillus*, *Descomyces*, *Penicillium*, *Phytophthora*, *Pythium*, *Rhizoctonia*, *Tricholoma*, *Torrendia*, *Thelephora*, and *Verticillium* [8].

Fungi are known to produce a plethora of natural products. About 47% (33,500 in a total of 70,000 metabolites obtained from microbes) of the bioactive secondary metabolites of microbial sources are from fungi [9]. Soil-derived fungi, to be specific, are regarded as an encouraging source of biologically active compounds owing to their skeletal structures and chemical complexities [10] that belong to various structural types, including aromatic compounds, amino acids, anthraquinones, butenolides, cytochalasins, macrolides, naphthalenones, pyrones, and terpenes [11]. Moreover, nearly one-third of fungal metabolites are from the genera *Aspergillus* and *Penicillium* [12]. 

Previous studies have reported that ethyl acetate extracts of the fungi *Aspergillus*, *Penicillium* and *Rhizopus* isolated from different sites of Malaysian forest soil showed potential antibacterial activity against *B. subtilis* and *E. coli* [13,14,15]. Recently, Alkhulaifi et al. (2019) reported that several fungal species isolated from Sultanate of Oman soil, identified as *A. flavus*, *A. terreus*, *A. athecius*, *F. chlamydosporum* and *F. nygamai*, showed significant antimicrobial activity against *C. tropicalis*, *C. glabrata*, *C. parapsilosis*, *C. albicans*, *L. acidophilus*, *S. gordonii*, *S. mutans* and *P. aeruginosa* [16]. A study by Kaur et al. (2015) reported that the compound methoxy-2,2-dimethyl-4-octa-4′,6′-dienyl-2H-naphthalene-1-one isolated from the fungus *Chaetomium globosum* showed significant anticancer activity against A549, MCF-7, PC-3 and THP-1 cancer cell lines [17].

*Aspergillus* is a ubiquitous and fast-growing fungus. The World Data Centre for Micro-organisms (WDCM) reports about 378 species of *Aspergillus*, of which approximately 180 fungal species are of pharmaceutical and commercial importance [18,19,20]. Due to its diversity, *Aspergillus* undoubtedly remains one of the most significant contributors to interesting secondary metabolites that exhibit anti-inflammatory, anticancer, antioxidant and antibacterial activities [21]. Sunil et al. (2014) reported that *Aspergillus oryzae* produces a high level of L-Glutaminas and potential anti-oncogenic activity against the MCF-7 cell line of human breast cancer [22].

Supattra et al. (2016) isolated *Neosartorya hiratsukae* and *Neosartorya pseudofischeri* from soil samples. They screened against several cancer cell lines, including HepG2, MCF-7, L929, HeLa, HT-29, L929, KB, Vero and P388 [23]. It was seen that the ethyl acetate extract of *N. hiratsukae* and *N. pseudofischeri* displayed the highest anticancer activity against the L929 cell line compared to the other cell lines. Such metabolites in fungal extracts have been recognized in cancer therapy because of their effectiveness in the creation of oxidative stress. It was recently found that stress signals (oxidative stress) in cancer cells can induce the imbalance in the equilibrium state of reactive oxygen species and free radicals within cells [24]. This mechanism can injure critical cellular components such as proteins, DNA and membrane lipids, and leading to cell death. These changes in the tumor cells by the effective metabolites finally induce programmed cell death in cancer cells [25].

Soil fungi are a relatively less studied community among the biogenetic components, and can be further explored to discover new products. Therefore, in the present study, fungi isolated from soil samples of Haliyal, Uttara Kannada, Karnataka, India. The soil samples were collected from the rhizospheric regions and screened for antimicrobial, antifungal and anticancer metabolites. The selected efficient strains were identified based on molecular characteristics. The crude extracts of the selected species were evaluated against human microbial pathogens and phytopathogens, and for their anticancer potential, by in vitro assessment against a breast cancer cell line. This investigation could help us to isolate novel compounds from soil fungi, which could be developed as better antibacterial and anticancer drugs.

Specific Objectives of the study:

1. Isolation, primary screening, and identification of fungal species isolated from rhizospheric soil;
2. Assessment of antimicrobial and antifungal activity of isolated fungal extract (AK-6) against human pathogens and phytopathogens;
3. Molecular characterization of potent fungal isolate AK-6. FT-IR and GC-MS analysis of the ethyl acetate extract of isolate AK-6;
4. Assessment of the anticancer activity of the ethyl acetate extract of AK-6 using the in vitro cytotoxic assay method against the MCF-7 breast cancer cell line;
5. Evaluation of apoptosis inducing efficacy of ethyl acetate extract from AK-6 against the MCF-7 breast cancer cell line by flow cytometric analysis.

## 2. Materials and Methods

### 2.1. Collection of Samples, Chemicals, Pathogens, and Cancer Cell Line

The rhizospheric soil samples were collected at Haliyal (latitude 15°19′50″ N and longitude 74°45′53″ E) Uttara Kannada, Karnataka, India, from the rhizospheric regions of different plants at a 5–10 cm depth using a steel borer. These samples were brought to the laboratory in sterilized polythene bags and stored at 4 °C for future work. Chemicals and reagents were obtained from HiMedia Laboratories; the human bacterial and yeast pathogens were procured from the Microbial Type Culture Collection (MTCC), Chandigarh, India. The phytopathogens were acquired from the Department of Plant Pathology, University of Agricultural Sciences (UAS), Dharwad, Karnataka, India. The MCF-7 cancer cell line (Human breast adenocarcinoma) was procured from the NCCS (National Centre for Cell Science) in Pune, India. In addition, camptothecin (Sigma Aldrich), fluorescein isothiocyanate annexin-V (Biosciences), and propidium iodide (Biosciences) were collected for anticancer studies.

### 2.2. Isolation of Fungi

The isolation of fungi from collected soil samples was done using the serial dilution technique. In total, 1 g of soil sample was dissolved in 9 mL of sterilized distilled water and diluted up to 5 fold (10^−5^). The aliquot of diluted soil was spread on PDA media, and plates were incubated for 3 to 7 days at 25 °C. After incubation, the grown fungal strain was subjected to subculture and pure culture techniques.

### 2.3. Morphological Characterization 

The pure colonies of fungal isolates were morphologically identified by observing color, mycelia, pigment production, and sporulation. In addition, the morphological, mycelia and spore structures were observed using a compound microscope (OLYMPUS CX23, Shanghai, China).

### 2.4. Extraction of Secondary Metabolites 

The isolate AK-6 obtained from the rhizospheric soil was selected to extract secondary metabolites. An isolated fungus was grown on a 250 mL conical flask containing potato dextrose broth for 21 days. The metabolites in the culture filtrate were extracted by adding an equal amount of ethyl acetate (1:1). Then, the culture filtrate with the ethyl acetate was transferred to a separating funnel. Further, it was kept for 24 h for the extraction of metabolites, as intermediate shaking of the culture filtrates with solvent enhances the extraction of compounds. The secondary metabolites of culture filtrate present on the upper layer of the separation funnel were collected and filtered to get the pure extract. The biomass of isolate AK-6 was ground in a pestle and mortar by adding 8–10 mL of ethyl acetate to extract the secondary metabolites. The suspension obtained was purified and filtered. The filtered suspension was poured into a watch glass and left to air dry for four h to evaporate the solvent. The metabolic extract left in the watch glass was scraped and collected in an eppendorf tube for future work [26].

### 2.5. Antimicrobial Activity of AK-6 Isolate against Human Pathogens

The potent isolate AK-6 was selected from the primary screening. The antimicrobial activity of the isolate AK-6 extract was determined against *Klebsiella pneumoniae* (MTCC 9238), *Escherichia coli* (MTCC 40), *Shigella flexneri* (MTCC 1457), *Bacillus subtilis* (MTCC 6633), *Staphylococcus aureus* (MTCC 6908) and yeast pathogen *Candida albicans* (MTCC 227) by the agar well diffusion method. Recently, a study has shown that *C. albicans* might produce substances that affect drug resistance in biofilm-forming *E. coli* isolated from blood. It was found that biofilms formed by a combination of species of *E. coli* and *C. albicans* showed tolerance to antimicrobial drugs. So, we studied *C. albicans* along with other bacteria [27,28]. Briefly, about 6 mm-diameter wells were made after thoroughly swabbing the pathogens mentioned above in individual petri dishes containing Muller–Hinton agar media (pH 7.4) [16]. Then, 25, 50, 75 and 100 μL of AK-6 extract was poured into the wells and streptomycin and amphotericin-B were used as standards for bacterial and yeast pathogens, respectively. After the incubation period at 37 °C, the antimicrobial activity was recorded by measuring the inhibition zones of respective concentrations of isolated AK-6 extract.

### 2.6. Antifungal Activity of Isolate AK-6 against Phytopathogens 

The antifungal activity of isolate AK-6 against phytopathogenic fungi such as *Sclerotium rolfsii* (Southern blot pathogen of chili), *Cercospora canenscens* (Cercospora leaf spot pathogen of mung bean) and *Fusarium sambucinum* (Dry rot pathogen of potato) was conducted using a dual culture method. Briefly, a colony of isolate AK-6 was placed in the middle of the Potato Dextrose Agar (PDA) plate. A fungal disc of pathogens was placed at both ends of the PDA plates. Then the plates were incubated at 28 °C. The antagonistic activity was examined at 3, 5, and 7 days after incubation by measuring the radius between the antagonist colony (R_2_) and phytopathogen (control) colony (R_1_). The antagonistic activity was calculated using the following formula [29]:

(1) $$\mathrm{Percentage}\;\mathrm{of}\;\mathrm{inhibition}\;\mathrm{of}\;\mathrm{growth}\;\mathrm{of}\;\mathrm{pathogen}\;=\frac{R_{1}-R_{2}}{R_{1}}\times 100$$

(R_1_ = Growth of fungal phytopathogens in control. R_2_ = growth of pathogens in dual culture plate).

### 2.7. Molecular Characterization of Isolate AK-6 

The DNA was extracted from the active culture of the isolate AK-6, and the 18S rRNA gene was amplified using primers. The chain reaction was scheduled with initial denaturation (25 cycles at 96 °C) for 5 min, hybridization (50 °C) for 30 sec, and final extensions (60 °C) for 1.5 min. Then the PCR products were subjected to electrophoreses on 1% agarose gel, where a 500-base pair DNA ladder was utilized as a size reference. The amplicons were purified and sequenced (Sanger Sequencing 3500 Series, Genetic Analyzer), and gene sequences were sought in the NCBI database via a BLAST web portal. Finally, the alignment and phylogenetic tree were constructed by the neighbor-joining method using the software MEGA version 7.0 [30].

### 2.8. PCR Amplification 

The PCR amplification of the total genomic DNA of isolate AK-6 was performed according to the method of Doyle and Doyle (1987) [31]. The regions of nrDNA (ITS1 5.8s and ITS2) were amplified using the primers 5′-TCCGTAGGTGAACCTGCGG-3′ and 5′-TCCTCCGCTTATTGATATGC-3′ [32]. Isolates of 18s rRNA sequences were submitted to the Gene Bank (NCBI, USA). After the NCBI BLAST analysis, the most similar fungal isolates were selected. The phylogenetic relationship of the isolate AK-6 was studied by constructing the phylogenetic tree using the neighbor-joining method. After the NCBI BLAST analysis, the most similar fungal isolates were selected. The phylogenetic relationship of the isolate AK-6 was studied by constructing the phylogenetic tree using the neighbor-joining method.

### 2.9. Fourier Transform Infrared Spectroscopy (FTIR) Analysis

The FTIR analysis of the ethyl acetate extract of isolate AK-6 was performed according to the methods of Bhat et al., 2022 [33]. The extract was dried using a thermostatted desiccator (at 45 °C for 24 h) and thoroughly mixed with potassium bromide (KBr). Finally, the functional groups were detected by making a thin disc of extract and KBr mixture, and analysis was performed in the range 400 cm^−1^ and 4000 cm^−1^ in an FTIR (Nicolet 6700, Thermo fisher Scientific, Waltham, MA, USA) instrument.

### 2.10. Gas Chromatography Mass Spectroscopy (GC-MS) Analysis

The GC–MS analysis of ethyl acetate extract of isolate AK-6 was carried out using a Shimadzu QP 2010 Ultra instrument, Kyoto, Japan. High-purity helium was employed as the carrier gas with a 1.2 mL/min flow rate. The electron ionization energy was 70 eV, while the ion source and interface temperature were set at 240 °C and 250 °C. A split–splitless injection with a split ratio of 1:10 was used at an injector temperature of 250 °C. A sample solution of 1 µL was injected. Column Rxi-5Sil MS (Thickness 0.25 µm, length 30 mm × 0.25 mm ID) was used. The oven temperature was initiated at 80 °C and was raised to 280 °C with a hold time of 2 min at 280 °C. Data computation was conducted using GC-MS solution data analysis software for the mass range 50–800 amu with a scan speed of 0.3 scan/s. Individual components were identified by comparing their mass spectra with those obtained from Adams, US National Institute of Standards and Technology (NIST, USA) and the WILEY mass spectra library [34].

### 2.11. Anticancer Activity by MTT Assay 

An MTT (cell viability) assay was -performed to evaluate the effects of the isolate AK-6 extract on cell proliferation. The MCF-7 cancer cell line (Human breast adenocarcinoma) was purchased from the NCCS (National Centre for Cell Science) in Pune, India. Briefly, the MCF-7 cells were cultured on 96-well (Thermo scientific) flat-bottom plates with a cell density of 20,000 cells/well and allowed to grow for 24 h. Then these cells were treated with 12.5, 25, 50, 100 and 200 µg/mL of ethyl acetate extract of Ak-6 for 24 h, and the MTT reagent was added to achieve a final concentration of 0.5 mg/mL. The camptothecin 5 μM/mL was used as a positive control and MCF-7 cell line without any treatment was considered as the untreated control. Then, the plates were incubated for 24 h at 37 °C in a 5% CO_2_ incubator. Further, light exposure was avoided by wrapping in aluminum foil, and it was incubated for three h. Then the MTT reduction product (formazan) was dissolved in 100 µL of DMSO with continuous shaking, and absorbance was read at 570 nm and 630 nm using an ELISA reader [35]. The cell viability was calculated using the below formula:

(2) $$\%\;\mathrm{Cell}\;\mathrm{viability}\;=\frac{\mathrm{OD}\;\mathrm{of}\;\mathrm{treated}\;\mathrm{cells}}{{\mathrm{OD}\;\mathrm{of}\;\mathrm{Untreated}\;\mathrm{cells}\;R}_{1}}\times 100$$

The IC_50_ value was determined using a linear regression equation, i.e., Y = Mx + C. Here, Y = 50, and the M and C values were derived from the viability graph.

### 2.12. Apoptosis Assay by Flow Cytometry 

The rate of cell death caused by the ethyl acetate extract of isolate AK-6 extract was studied against MCF-7 cancer cell line. The cultured MCF-7 cells were taken in 96-well plates with a density of 0.5 × 106 cells/2 mL and incubated at 37 °C in a CO_2_ incubator for 24 h. Further, the cells were treated with IC50 concentrations of ethyl acetate extract of isolate AK-6 for 24 h. The control trial was performed in 2 mL of culture media without treatment of the AK-6 ethyl acetate extract. At the treatment’s end, the cells were subjected to phosphate-buffered saline (PBS) washing. Then the PBS was discarded, and 200 µL of the trypsin–ethylenediamine tetra-acetic acid (EDTA) was amended and kept for incubation for 3 to 4 min at 37 °C. Further, 2 mL of culture medium was added and cells were harvested directly into polystyrene tubes (12 × 75 mm), and centrifugation was performed at 300 rpm for 5 min at 25 °C. Finally, the supernatant was removed and subjected to PBS washing. In total, 5 mL of fluorescein isothiocyanate (FITC) and annexin V were added to create a pellet by gentle vertex and incubated in dark conditions for 15 min at 25 °C; finally, 5 mL of propidium iodide (PI) with 400 mL of 1× binding buffer was added, and analysis was performed by flow cytometry(BD FACS Calibur, Flow Cytometer) [36]. 

### 2.13. Statistical Analysis 

The antimicrobial and anticancer experiment results are expressed as means ± standard deviation (SD, for each group *n* = 3) to prepare graphs. In addition, 2-way ANOVA and Tukey’s multiple comparisons evaluated statistical significance at *p* < 0.05 between the different antimicrobial groups.

## 3. Results

### 3.1. Isolation of Fungi and Primary Screening

**Figure 1:**
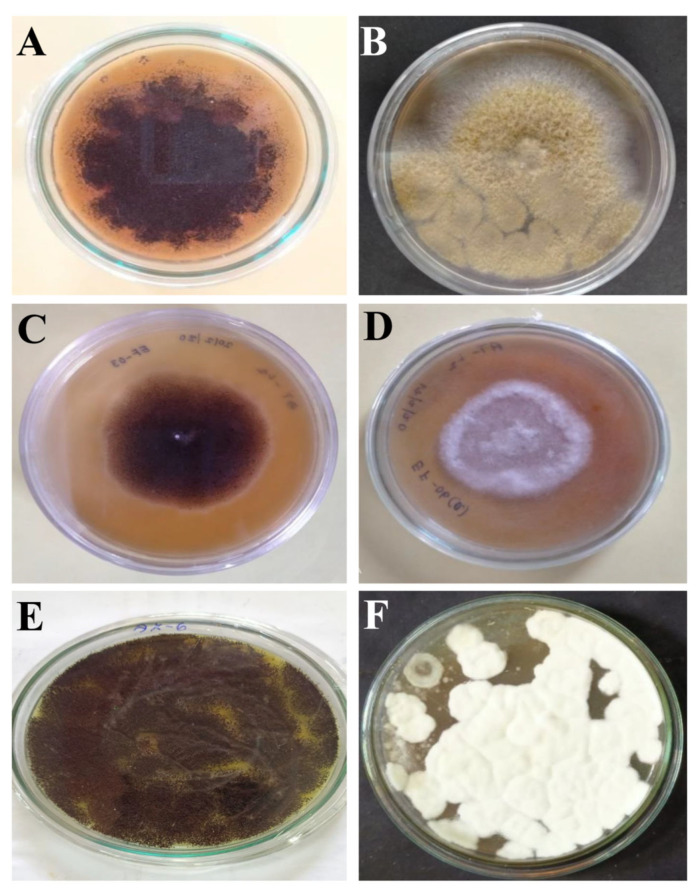
Isolation of different fungi from the collected soil samples. (**A**) AK-1, (**B**) AK-2, (**C**) AK-3, (**D**) AK-4, (**E**) AK-6, and (**F**) AK-7.

In the present study, six fungal isolates were recovered from soil samples collected from Haliyal, Uttara Kannada, Karnataka. Among the six fungal isolates (AK-1, 2, 3, 4, 6, 7) (Figure 1), AK-6 showed maximum activity for primary screening. So, it was further used to evaluate the antimicrobial, antifungal and anticancer activity.

### 3.2. Morphological Characterization

**Figure 2:**
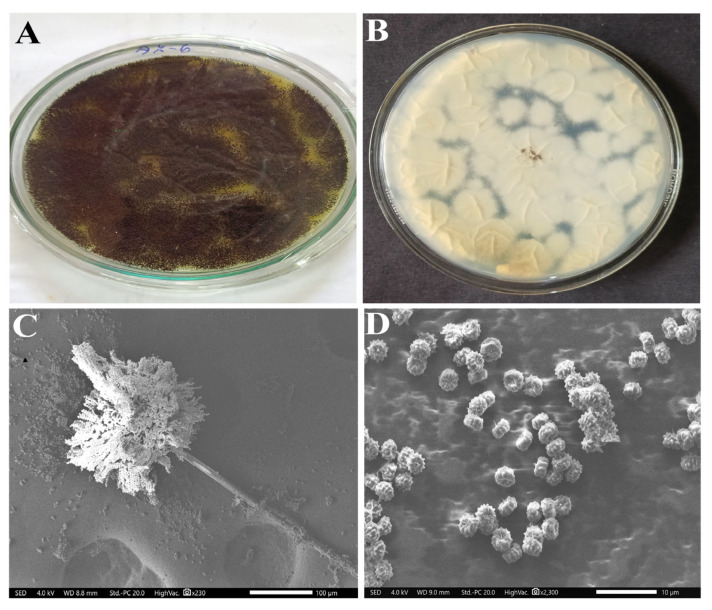
Morphological characterization of isolate AK-6. (**A**) aerial mycelium, (**B**) substrate mycelium, (**C**) scanning electron microscope image showing conidiophores and (**D**) scanning electron microscope image showing spore morphology.

The most potent fungal isolate, AK-6, was selected for the morphological characterization. The morphologically isolated AK-6 showed *Aspergillus* sp. Characteristics, such as a milky white-colored biomass and complete black-colored sporulation on maturity (Figure 2A,B). In addition, the microscopic study revealed that prominent mycelia with emerging conidiophores ended with numerous metula and phialides (Figure 2C). The phialides showed the number of conidia bearing rounded spores, confirming the characteristic features of *Aspergillus* sp. (Figure 2D). Finally, isolate AK-6 was morphologically identified as *Aspergillus* sp. The isolate AK-6 showed good growth performance in potato dextrose broth.

### 3.3. Antimicrobial Activity against Human Pathogens

**Figure 3:**
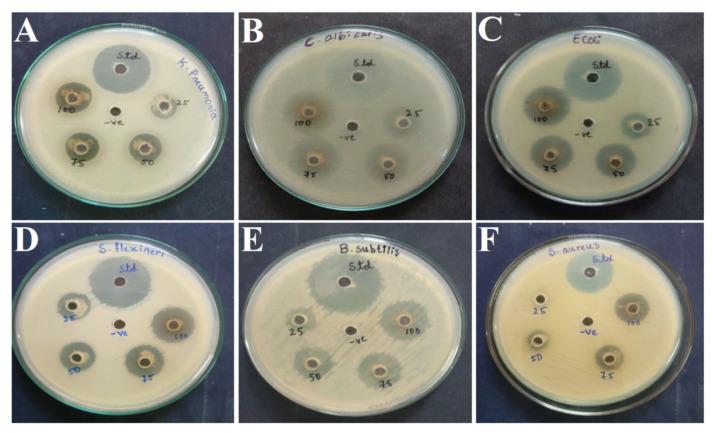
Antimicrobial activity of ethyl acetate extract of isolate AK-6. (**A**) *K. pneumonia*, (**B**) *C. albicans*, (**C**) *E. coli*, (**D**) *S. flexneri*, (**E**) *B. subtilis* and (**F**) *S. aureus*.

**Figure 4:**
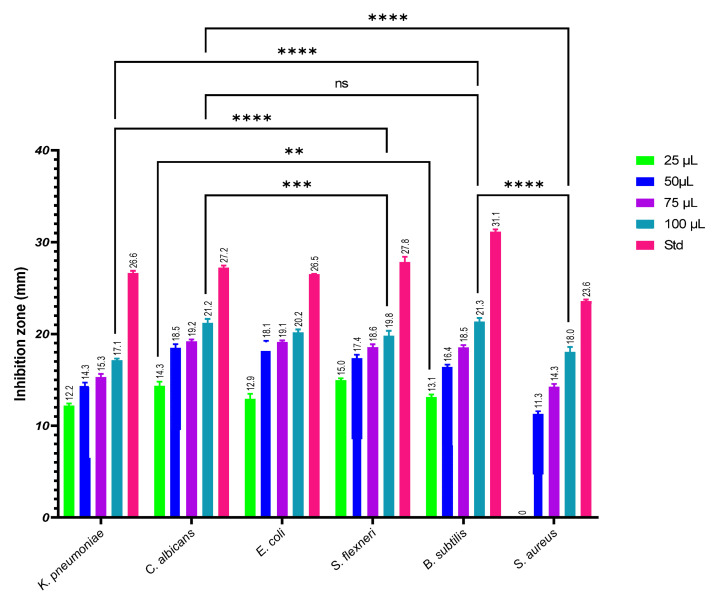
Graphical representation of the antimicrobial activity of ethyl acetate extract isolate AK-6 against pathogenic microorganisms. Two-way ANOVA with Tukey’s multiple comparisons was performed to identify group differences. *p* < 0.01 (**), *p* < 0.001 (***), and *p* < 0.0001 (****). ns: not significant.

The primary screening of the six isolated fungi was performed by the cross streak method, and the isolate AK-6 recorded maximum inhibition. Further, isolate AK-6 was selected for the secondary screening to determine its antimicrobial activity by the agar well diffusion method using ethyl acetate extract against six human pathogens (Figure 3A–F), namely, *Klebsiella pneumonia*, *Candida albicans*, *Esherichia coli*, *Shigella flexneri*, *Bacillus subtilis* and *Staphylococcus aureus*. The results show a maximum inhibition zone at 100 µL against all tested pathogens, and lesser inhibition of their growth with 25 µg/mL, with the highest inhibition zone at 22.35 ± 0.20 mm against *Bacillus subtilis* followed by 21.30 ± 0.26 mm against *Candida albicans*. In comparison, the lowest antimicrobial activity was exhibited against *Klebsiella pneumonia* with an inhibition zone of 16.36 ± 0.14 mm at 100 µL. Moderate antimicrobial activities, with inhibition zones of 20.57 ± 0.12 mm, 20.43 ± 0.19 mm and 18.39 ± 0.03 mm against *Shigella flexneri*, *Esherichia coli*, and *Staphylococcus aureus*, respectively, was observed at 100 µL. The antimicrobial potential of the ethyl acetate extract of isolate AK-6 was statistically significant among the groups, as depicted in Figure 4 and Table 1. The standard streptomycin exhibited 26.6 ± 0.23, 26.5 ± 0.47, 27.8 ± 0.38, 31.1 ± 0.41, and 23.6 ± 0.29 mm of inhibition against *Klebsiella pneumonia*, *Esherichia coli*, *Shigella flexneri*, *Bacillus subtilis*, and *Staphylococcus aureus*, respectively. An inhibition of 27.2 ± 0.39 mm was observed when using standard amphotericin B against *Candida albicans*. 

### 3.4. Antifungal Activity against Phytopathogens

**Figure 5:**
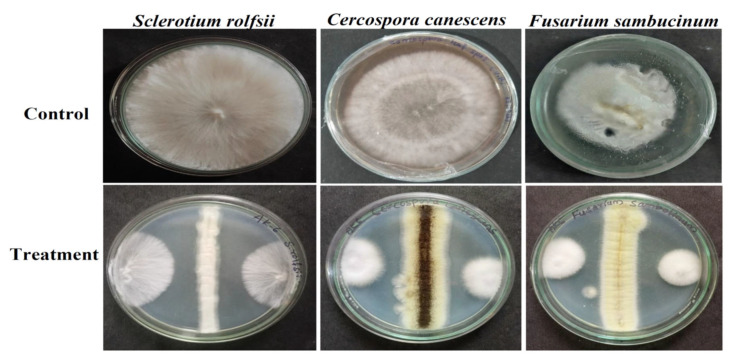
Antifungal activity of isolate AK-6 assessed by dual culture assay against phytopathogens.

**Figure 6:**
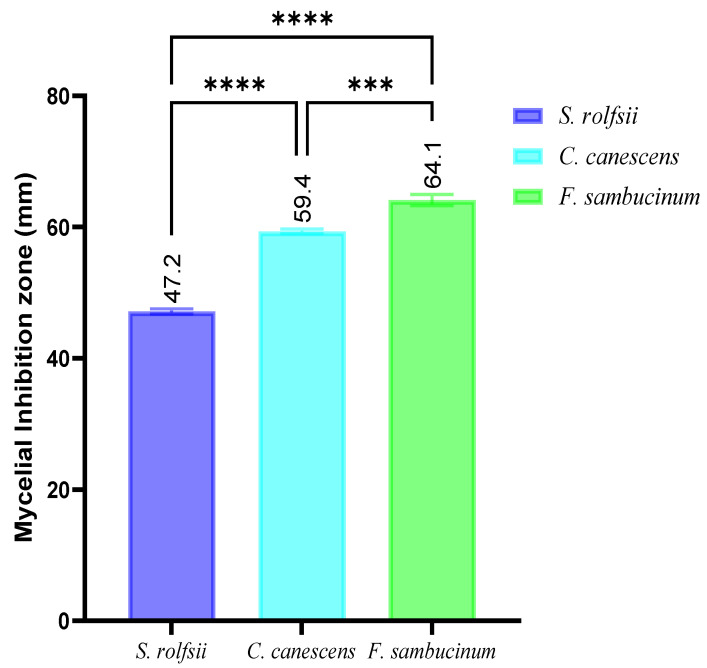
Graphical representations of antifungal activity of isolate AK-6 against phytopathogens assessed by dual culture assay. Two-way ANOVA with multiple comparisons was accomplished to identify group differences. *p* < 0.001 (***), and *p* < 0.001 (****).

The antifungal activity of isolate AK-6 was assessed by the dual culture method against three phytopathogens, namely, *Sclerotium rolfsii*, *Cercospora canenscens*, and *Fusarium sambucinum* (Figure 5). The results show 47.2% inhibition against *Sclerotium rolfsii*, 59.4% against *Cercospora canenscens* and 64.1% against *Fusarium sambucinum*. The highest antifungal activity was exhibited against *Fusarium sambucinum*, and the most negligible action against *Sclerotium rolfsii*. All the results have been compared with the respective phytopathogens grown in control plates. A graphical representation of the antifungal activity of isolate AK-6 is depicted in Figure 6.

### 3.5. Molecular Characterization of Isolate AK-6

**Figure 7:**
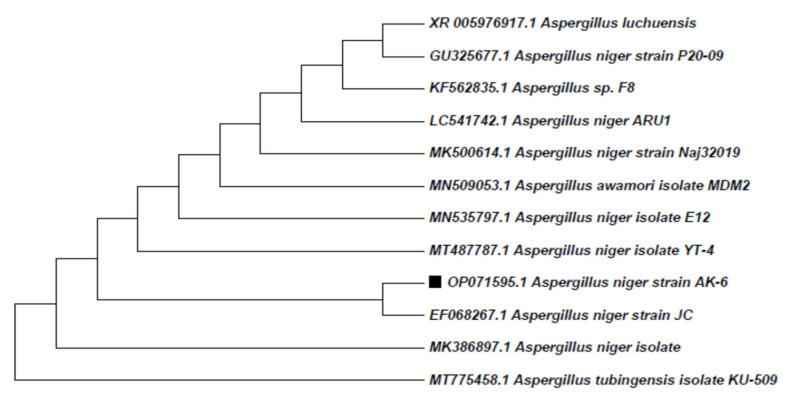
Dendrogram showing the phylogenetic relationship of *Aspergillus niger* strain AK-6.

The genomic DNA of isolate AK-6 was extracted and analyzed by 18S rRNA gene sequencing. For identifying micro-organisms up to the species level, the 18S rRNA gene sequencing is widely used in the construction of phylogenies because of the slow rate of evolution at that gene region [37]. A total length of 956 base pairs was submitted to the NCBI gene bank with the accession number OP071595. This sequence was later subjected to a search for close relatives through nucleotide blast (BLASTN) in NCBI. The analysis of the 18S rRNA gene sequence of the AK-6 revealed 99.79% sequence similarity with the *Aspergillus niger* strain JC. Additionally, a phylogenetic tree was reconstructed through the neighbor-joining method with the obtained gene sequences. The phylogenetic tree analysis revealed that isolate AK-6 fell within the cluster of the genus *Aspergillus* and formed a branch with the *Aspergillus niger* strain JC, as represented in Figure 7. Furthermore, the molecular characterization shows that isolate AK-6 belongs to *Aspergillus niger* (*Aspergillus niger* strain AK-6).

### 3.6. FTIR Analysis

**Figure 8:**
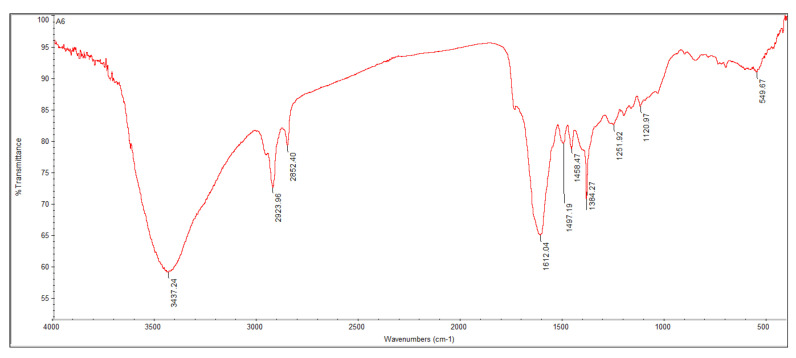
FTIR analysis of ethyl acetate extract of *Aspergillus niger* strain AK6.

FTIR analysis of the *Aspergillus niger* strain AK-6 shows absorption peaks at 3437, 2923, 2852, 1612, 1497, 1458, 1384, 1251, 1120 and 549 cm^−1^, which represent different functional groups (Figure 8). The medium and sharp absorption peaks at 3437 and 2923 cm^−1^ correlate with O–H stretching alcohol and C–H stretching alkane. The sharp, strong peaks at 2852 and 1612 cm^−1^ represent C–H stretching alkane and the α, β-unsaturated ketone. The absorption peaks at 1497 and 1458 cm^−1^ depict N–O stretching nitro compound and C–H bending alkane. The presence of S=O stretching sulfate and C–N stretching alkyl aryl ether have been confirmed by the existence of absorption peaks at 1384 and 1251 cm^−1^, respectively. The weak and medium peaks at 1120 and 549 cm^−1^ correspond with C–O stretching aliphatic ether and C–I stretching halo compound (Table 2).

### 3.7. GC-MS Analysis

**Figure 9:**
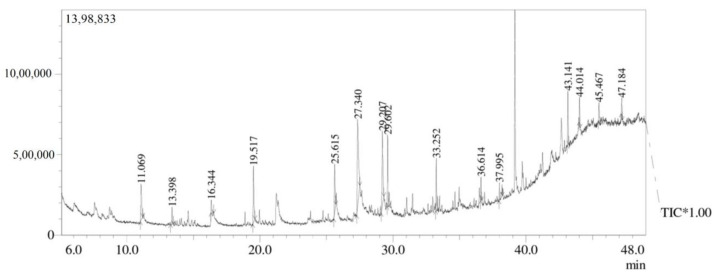
GC-MS chromatogram of ethyl acetate extract of *Aspergillus niger* strain AK-6.

**Figure 10:**
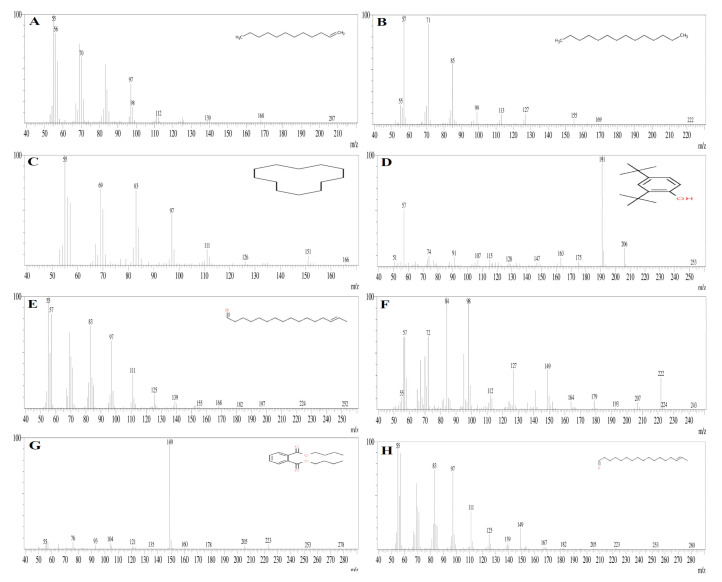
Mass spectra of individual metabolites found in ethyl acetate extract of the *Aspergillus niger* strain AK-6. (**A**) 1-Dodecene, (**B**) tetradecane, (**C**) cyclodecane, (**D**) 2,4-di-tert-butylphenol, (**E**) E-14-hexadecenal, (**F**) N-didehydrohexacarboxyl-2,4,5-trimethylpiperazine, (**G**) dibutyl phthalate and (**H**) E-15-heptadecenal.

**Figure 11:**
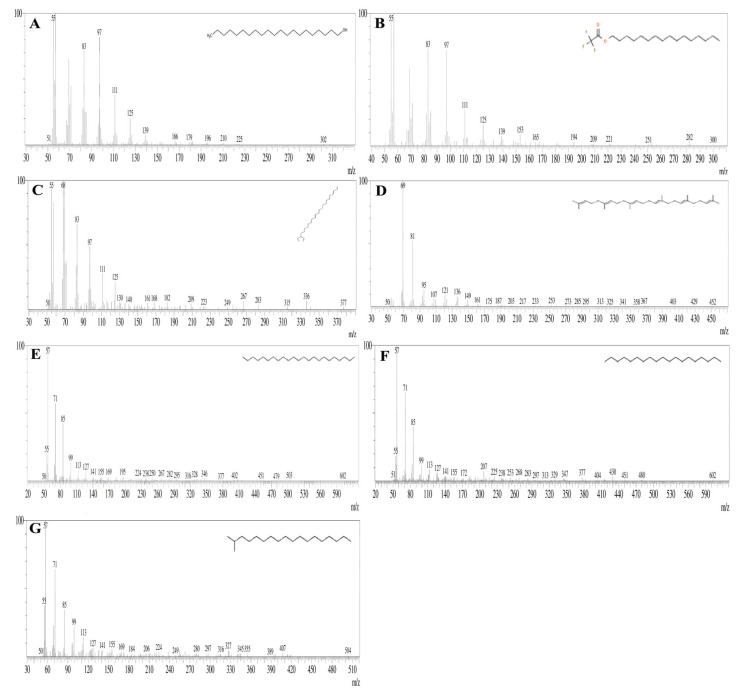
Mass spectra of individual metabolites found in ethyl acetate extract of the *Aspergillus niger* strain AK-**6**. (**A**) 1-Heneicosanol, (**B**) trifluroacetoxy hexadecane, (**C**) cyclopentane, heneicosyl, (**D**) squalene, (**E**) tricosane, (**F**) nonadecane and (**G**) octadecane 2 methyl.

The present study analyzed the ethyl acetate extract of Aspergillus niger strain AK-6 for bioactive compounds via a GC-MS analysis. The analyses revealed the presence of 15 compounds, which were identified from the chromatogram. The chromatogram is shown in Figure 9, while the chemical constituents with their retention time (RT), molecular weight, molecular formula, and peak area are presented in Table 3. The GC-MS analysis identified the following compounds: 1-dodecene, tetradecane, cyclodecane, 2, 4-ditert-butyl-phenol, e-14-hexadecenal, n-didehydrohexacarboxyl-2,4,5-trimethyl piperazine, dibutyl phthalate, e-15-heptadecenal, 1-heneicosanol, trifluroacetoxy hexadecane, cyclopentane, heneicosyl, squalene, tricosane, nonadecane, and octadecane 2-methyl. Table 3 displays the molecular structures of some important constituents found in the ethyl acetate extract of the *Aspergillus niger* strain AK-6. The compounds from the GC-MS analysis were identified based on RT and peak area. The first compound 1-dodecane was identified with a shorter RT (11.069), and the last octadecane, 2-methyl was identified with by far the longest RT (47.34), while n-didehydroexacarboxyl-2,4,5-trimethylpiperazine showed the highest percentage peak area (23.82%) and peak height (14.19%). Among these 15 compounds, tetradecane, 2,4-ditert-butylphenol, n-didehydroexacarboxyl-2,4,5-trimethylpiperazine, dibutyl phthalate and nonadecane were identified as the major compounds (Figure 10). The mass spectra of individual compounds obtained from the GC-MS analysis of the ethyl acetate extract of *Aspergillus niger* strain AK-6 are displayed in Figure 10A–H and Figure 11A–G.

### 3.8. Anticancer Activity: Cell Viability by MTT Assay

**Figure 12:**
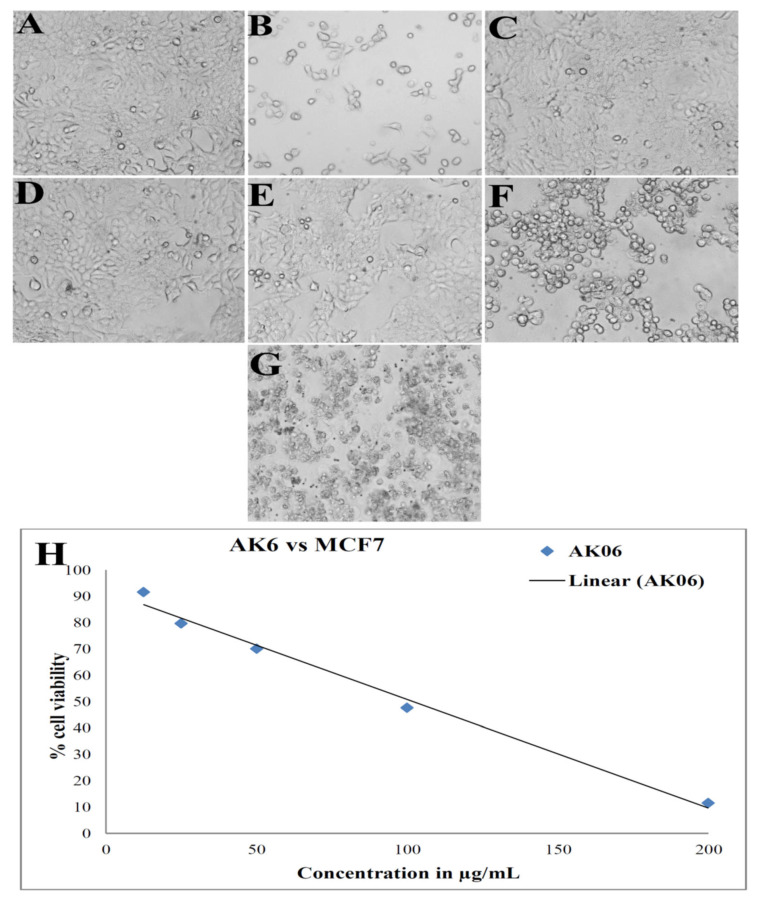
Anticancer activity (MTT assay) of ethyl acetate extract of the *Aspergillus niger* strain AK-6 against the MCF-7 cell line. (**A**) Untreated, (**B**) positive control treated, (**C**) 12.5 μg/mL, (**D**) 25 μg/mL, (**E**) 50 μg/mL, (**F**) 100 μg/mL, (**G**) 200 μg/mL, (**H**) graphical representation of anticancer activity of the *Aspergillus niger* strain AK-6 extract.

The ethyl acetate extract of the *Aspergillus niger* strain AK-6 was treated with different concentrations against the human breast cancer cell line, MCF-7, to test cell viability/cytotoxicity using an MTT assay. Morphological changes exhibited cell shrinkage, and rounding at varying degrees was evident in the images, emerging in a dose-dependent manner (12.5, 25, 50, 100 and 200 μg/mL) (Figure 12A–G). The viability of cancer cells at 12.5, 25, 50, 100, and 200 μg/mL gavce values of 91.60%, 79.68%, 70.05%, 47.65% and 11.25%, as represented in Figure 12H. There was no change in the cell viability for untreated cell lines. Therefore, the test result exhibits a concentration-dependent decrease in the viability of the MCF-7 cell line. The positive control, camptothecin, showed 44.46% cell viability at the treated concentration, and 100% viability was noted in the untreated cell line. The analysis of the MCF-7 cell line treated with the ethyl acetate extract of the *Aspergillus niger* strain AK-6 showed significant growth-inhibitory potential, with an IC_50_ value at a concentration of 102.01 μg/mL compared to the positive control, camptothecin (20 µM/mL), with an IC_50_ concentration at 5 µM/mL equivalent to the 1.96 µg/mL used for the study, after incubation for 24 h.

### 3.9. Apoptosis Assay

**Figure 13:**
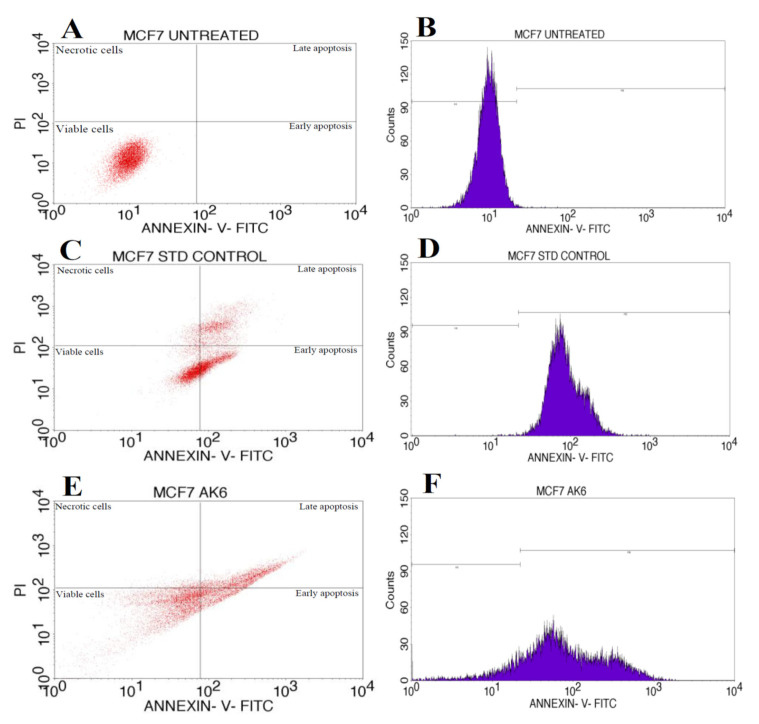
Flow cytometric analysis of ethyl acetate extract of the *Aspergillus niger* strain AK-6 against the MCF-7 cell line. Quadrangular plot of Annexin V/PI expression on MCF-7 cells: (**A**) untreated control group, (**B**) Cell cycle of MCF-7 cell line in untreated control group. Quadrangular plot of Annexin V/PI expression on MCF-7 cells: (**C**) positive control treated group. (**D**) Cell cycle of MCF-7 cell line positive control treated group. Quadrangular plot of Annexin V/PI expression on MCF-7 cells: (**E**) MCF-7 cell line treated with ethyl acetate extract of the *Aspergillus niger* strain AK-6. (**F**) Cell cycle of MCF-7 cell line treated with ethyl acetate extract of the *Aspergillus niger* strain AK-6.

The ethyl acetate extract of the *Aspergillus niger* strain AK-6 with an IC_50_ concentration of 102.01 μg/mL was used for the AnnexinV/PI expression study on the MCF-7 cell line (Figure 13), which shows the proportion of cells that underwent apoptosis and necrosis, and the presence of viable cells. The ethyl acetate extract of the *Aspergillus niger* strain AK-6 treated with the MCF-7 cell line became apoptotic after 24 h, with 17.3% early apoptosis, 26.43% late apoptosis, 53.11% healthy cells and 3.16% necrosis, suggesting a decrease in cell viability. The untreated MCF-7 cells showed no apoptosis, and 100% healthy cells were observed. In the positive control, 31.93% early apoptosis, 21.33% late apoptosis, 42.77% healthy cells and 3.97% necrosis were observed (Table 4).

## 4. Discussion

Natural products and their derivatives have been widely used for human health since the 1940s, especially for cancer and microbial infections [38]. Since penicillin’s discovery, several thousand natural, synthetic, and semi-synthetic antibiotics have been used. Many of them are isolated from bacteria and fungi. For example, fungi, including *Penicillium*, *Aspergillus*, *Fusarium*, *Cladosporium* and yeasts, produce enzymes and secondary metabolites with antibiotic activity [39]. These antibiotics produced by fungi, such as fusidic acid, cephalosporin, and penicillin, have been widely used to treat many infectious diseases, and vancomycin has been treated by the bacterium known as *Amycolatopsis orientalis*, which is a true antibiotic [40,41]. Unfortunately, the need for new antibiotics is increasing due to the emergence of drug-resistant pathogens and many synthetic drugs causing several side effects. *A. niger* strains produce various secondary metabolites, but only ochratoxin A can be observed as a mycotoxin [42].

Soil microorganisms are the most critical group on Earth, producing active biological components possessing abundant activities beneficial to humankind. Mainly, fungal species are potential producers of bioactive compounds. More than 500 antibiotics are discovered yearly; however, almost 60% are obtained from the soil, this thereby representing the most critical reservoir for novel antibiotics with pharmaceutical and biological activity [43]. An investigation by Sohail et al. (2014) reported the antimicrobial potential of acetonitrile and n-hexane extracts of the *R. stolonifer* against some fungal and bacterial pathogenic strains [44]. In this study, the acetonitrile extract of *R. stolonifera* inhibited several fungal pathogenic strains such as *A. niger*, *A. oryzae*, *C. albicans*, *P. digitatum* and *F. oxysporum*, and the bacterial pathogens *P. aeruginosa*, *E. coli*, *S. aureus*, *S. aureus* (methicillin-resistant) and *S. aureus* (vancomycin-resistant), more effectively than the n-hexane extract. Rabiyathul et al. (2017) studied the antibacterial activities of ethyl acetate extracts of six *Trichoderma* sp. isolated from marine mangrove rhizosphere soil. All the *Trichoderma* sp. significantly inhibited the growth of Gram-positive bacteria *S. aureus*, followed by the Gram-negative bacteria *E. coli*. Minimum inhibition was noted against *P. aeruginosa* [45].

Furthermore, soil fungi have been treated as a potential means of inhibiting the growth of various cancer cell lines. Di-2-ethylhexyl phthalate and 1,8-dihydroxy-3-methoxy-6-methyl-anthraquinone, isolated from extracts of *Eurotiumtonophilum* and *Drechslera rostrata*, expressed anticancer activities against colon, cervical, larynx and hepatocellular cancers [46]. Furthermore, Salehi et al. (2018) isolated the polysaccharide compounds from *Fusarium* sp. and exhibited an excellent cytotoxicity effect on the Lymphoblastoid and HeLa cell lines [47].

In our study, the cultured fungal isolates were identified based on the culture morphology and spore characteristics by referring to the manual of the pictorial atlas of soil and seed fungi. Of the six fungal isolates, three species belong to the genera of *Aspergillus* (AK-1, AK-2 and AK-6), and one species belongs to *Collettotricum* sp. (AK-3), *Pythium* sp. (AK-4) and *Penicillium* sp. (AK-7) each. Similarly, Wibowo et al. (2017) observed that, among the 17 isolates, *Aspergillus* as the most dominant fungal genus, followed by *Penicillium* and *Rhizopus,* in the forest soil of Malaysia [13]. Melo et al. (2018) found *Penicillium* and *Trichoderma* to be dominant among the 28 fungal strains isolated from the soils of Cerrado (Brazilian Savanna) [48]. 

In this study, the selected fungal AK-6 isolate was grown in a PDB medium for 21 days. After incubation, the crude extracts containing the metabolites were separated from the fermented medium with ethyl acetate. This was followed by screening for their antimicrobial potential against six human pathogens, three Gram-negative bacteria pathogens, namely, *Escherichia coli*, *Klebsiella pneumonia* and *Shigella flexineri*, two Gram-positive bacteria, *Staphylococcus aureus* and *Bacillus subtilis*, and one yeast pathogen, *Candida albicans*, by the well diffusion method. Additionally, its activity against phytopathogenic fungi, namely, *Fusarium sambucinum*, *Cercospora canenscens*, and *Sclerotium rolfsii*, was assessed using the dual culture method. This demonstrated the concentration-dependent antimicrobial activity against the tested microbes, with higher microbial growth inhibition when using 100 µg/mL of ethyl acetate extract of *Aspergillus niger* strain AK-6. Furthermore, it exhibited significant antimicrobial potential against the Gram-positive bacteria *B. subtilis*, followed by *C. albicans* and *E. coli*, whereas its activity was weakest against *S. aureus* and *K. pneumonia*. Similar to the present work, Makut and Owolewa (2011) reported that the antibacterial activity of *Aspergillus niger* and *Penicillium* sp. against *E. coli*, *P. aeruginosa* and *S. aureus* was observed with different zones of inhibition, due to the presence of potential secondary metabolites that cause enzyme inhibition and DNA damage in the pathogens, which leads to the lowering of cellular homeostasis and finally causes cell death [49]. In another study, Wibowo et al. (2017) reported that *Penicillium* and *Aspergillus* isolated from the forest soil showed the maximum antibacterial activity against Gram-positive and Gram-negative bacterial pathogens [13]. The antimicrobial action of any drug depends on its metabolite profile and the virulence of the pathogens. The different structural organizations of microbial pathogens play a significant role in their defense mechanisms. For example, in the teichoic acid, phospholipids, lipoteichoic acid and peptidoglycan proteins in Gram-positive (*Staphylococcus aureus*, *Bacillus subtilis*) and Gram-negative (*Escherichia coli*, *Klebsiella pneumonia*, *Shigella flexineri*) bacteria, the positions of peptidoglycan (between the cell membrane) are different. The outer layer can restrict the invasion or action of drugs and make the Gram-negative bacteria more resistant [13]. The primary cell wall components of *Candida albicans* are β-glucans (β-1, 3 and β-1, 6 linkages) (40 to 60%), minor chitin (up to 9%) and proteins (6 to 25%). These components give great rigidity to the cells [17]. 

Further, the antifungal efficacy of the *Aspergillus niger* strain AK-6 was assessed via a dual culture assay against three selected phytopathogens. The results show the highest activity against *Fusarium sambucinum* and the lowest antifungal activity against *Sclerotium rolfsii.* Moderate antifungal activity was exhibited against *Cercospora canescens*. The present study correlates with the report of Zulqarnain et al.; in their research, a different class of bioactive metabolites from *Aspergillus* sp. showed antifungal potential against different phytopathogens [50]. The antifungal activity was exhibited due to the metabolites, which cause physical decay in pathogenic fungal tissue that causes membrane leakage, as well as the disruption of growth of mycelia, which leads to a reduction in cellular metabolism and proliferation [51]. Marei et al. (2018) reported that changes in the fungal cell wall caused by antifungal metabolites alter the permeability of the cell membrane and lower cellular respiration, which causes the complete degradation of mycelia and inhibits the further growth of fungal pathogen [52].

The selected *Aspergillus niger* strain AK-6 fungus was identified by 18S rRNA gene sequencing. The fungal sequence of 956 base pairs with the accession number OP071595 was blasted through nucleotide blast (BLASTN) in the NCBI database to find the closely related fungal species. From the analyzed data, the 18S rRNA gene sequence of the AK-6 revealed 99.79% sequence similarity with the *Aspergillus niger* strain JC. Further, a phylogenetic tree was reconstructed through the neighbor-joining method with obtained gene sequences. The phylogenetic tree analysis revealed that the *Aspergillus niger* strain AK-6 fell within the cluster of the genus *Aspergillus,* and formed a branch with the *Aspergillus niger* strain JC, as represented in Figure 7. The 18S rRNA gene sequencing was the most promising mode of molecular characterization, enacted to identify the fungal isolates by amplifying 18S rRNA, which facilitates the phylogenetic variation among the fungal isolates [53]. Similarly, Waing et al. (2015) identified five fungal species, *Aspergillus eucalypticola*, *Aspergillus fumigatus*, *Colletotrichum gloeosporioides*, *Fusarium oxysporum* and *Penicillium echinulatum,* by ITS sequences and BLAST analysis [54]. 

FTIR analysis is the most widely utilized technique for identifying functional groups, and is the primary means of confirmation of metabolites present in test samples. It also helps in identifying bioactive metabolites [55]. The FTIR characterization of *Aspergillus niger* strain AK-6 suggested the presence of various functional groups, such as alkanes, carboxylic acids, ketones, hydroxyl groups, alcohol, sulfate and ethers. The major FTIR peaks of *Aspergillus niger* strain AK-6 represent bioactive compounds with various biological activities (antimicrobial, antifungal and anticancer). In correlation with the present study, the FTIR analysis of the *Aspergillus niger* strain depicted different functional groups known as effective compounds against human pathogens, including cancer cells and phytopathogens [56]. In another study, FTIR analysis of the fungal extract revealed that the different functional groups identified as carboxylic acids, sulfate, ketones and nitro compounds can have various medical and agricultural applications [57].

The chromatogram of the GC-MS analysis of the ethyl acetate extract of *Aspergillus niger* strain AK-6 revealed the presence of 15 compounds that might be involved in the above-described bioactivities. Among these compounds, N-didehydroexacarboxyl-2,4,5-trimethylpiperazine showed the highest peak areas of 23.82%. It was identified by Kanjana et al. (2019) as an antimicrobial and antioxidant, supporting the present analysis [26]. Similarly, the compound dibutyl phthalate was observed in this current study with a retention peak of 14.65%, and this was reported by Sharma et al. (2022) as an antibacterial, while Madhusudan et al. (2023) said that it eliminated tumor cells by regulating the cellular activity of caspase-3/CPP32, and induced apoptosis of leukemia cell lines [58,59]. Supardy et al. (2014) demonstrated the antibacterial activity of e-15-heptadecenal, and Skanda et al. (2021) described the antioxidant, anti-inflammatory, and anticancer properties of 2,4-ditert-butyl-phenol [34,60]. Dodecane obtained from the extract *Penicillium brasilianum* (MH198044) was reported by Mishra et al. (2017) from *Aspergillus clavatonanicus* as a growth inhibitor of plant and human pathogens [61]. Shamili et al. (2019) and Wijayanti et al. (2022) studied the antimicrobial potentials of the bioactive compounds tetradecane and nonadecane [62,63]. The compounds hexadecane, heneicosyl, and 1-heneicosanol have been reported in previous studies, and have shown antimicrobial and anticancer activities [17]. Li et al. (2023) documented that 1-dodecane, tetradecane, and octadecane-2-methyl have antimicrobial, antifungal and antitumor properties [64].

In our study, the ethyl acetate extract of *Aspergillus niger* strain AK-6 expressed a significant anticancer activity against the MCF-7 cancer cell line with an IC_50_ of 102.01 μg/mL. These results can be compared with the observed anticancer activity of the extract of *Chaetomium globosum* against MCF-7 cell lines, with a 147.87 μg/mL IC_50_ value, and which also caused a significant reduction in the viability of the tested cell line [65]. Likewise, Abutaha et al. (2018) reported that the crude extract of *Penicillium crustosum* showed significant anticancer activity against MCF-7, with a moderate IC_50_ value of 126 μg/mL [66]. Furthermore, Yodsing et al. (2018) observed the anticancer activity of an *Aspergillus aculeatus* crude extract against the growth of breast cancer cells. Further, they observed that the compounds secalonic acid D, variecolin, and variecolactone isolated from *Aspergillus aculeatus* showed potential anticancer activity against human breast cancer (MCF-7) [67]. Recently, Dhayanithy et al. (2019) reported that the culture filtrate extract of *Chaetomium nigricolor* showed anticancer activity against MCF-7 cell lines [68]. The anticancer efficacy of fungi was found to be mainly influenced by the bioactive secondary metabolites, which show cytotoxicity in cancer cells. With reference to the previous study, Mousa et al. (2021) reported that dibutyl phthalate and octadecane, which were identified in the extract of *Alternaria tenuissima,* exhibited potent cytotoxicity against the hepatocellular carcinoma cell line (HepG2) [69]. In another study, Sajna et al. (2020) described the role of antiproliferative metabolites derived from *Aspergillus unguis* AG 1.1 (G), which caused a significant reduction in the proliferation and viability of MCF-7, A-431, and COLO-205 cancer cell lines by causing DNA lesions within the cancer cells, which lead to DNA damage, arrest of the cell cycle, and finally cell death [70]. In the present study, different bioactive metabolites from the ethyl acetate extract of the *Aspergillus niger* strain AK-6 might be involved in reducing the viability of the tested MCF-7 cancer cell line. It was also shown by the study of Ayswarya et al. (2022) that the active metabolite 2, 4-ditert-butylphenol had an antiproliferation effect against the MCF-7 cell line, manifested by the induction of apoptotic genes, which caused DNA fragmentation and cell death [71]. Kaur N et al. (2020) reported that the GC-MS analysis of the fungus *Chaetomium globosum* showed the presence of e-15-heptadecenal and e-14-hexadecenal, which exhibited antiproliferative potential against different cancer cell lines [17]. Therefore, to emphasize the other mechanisms exerted, we carried out an Annexin V-FITC/PI apoptosis detection assay.

Apoptosis induction was confirmed through the Annexin V-FITC/PI apoptosis detection assay, which is extensively used to discriminate living cells from those undergoing early and late apoptosis. It was noted that the ethyl acetate extract of the *Aspergillus niger* strain AK-6 led to a strong shift to early and late apoptotic cell populations, which indicates that the ethyl acetate extract of the *Aspergillus niger* strain AK-6 caused the apoptosis cell death mode. The induction of the apoptotic signaling pathways to trigger cancer cell death is the primary mechanism of most anticancer drugs. The ethyl acetate extract of the *Aspergillus niger* strain AK-6 showed 43.73% apoptosis compared to the positive control, camptothecin, with 53.26% apoptosis, while 3.16% necrosis was shown, and therefore cell viability was decreased. Similar results have also been reported by Hendy et al. (2023), who reported that the L-methionine γ-lyase isolated from *Aspergillus fumigates* led to maximum susceptibility and increased apoptosis in Hep-G2 and HCT cells [72]. Tomikava et al. (2000) reported that Rasfonin, a powerful natural drug isolated from *Talaromyces* sp. 3656-A1, exhibited increased caspase-dependent apoptosis and also activated necroptosis [73]. Another study by Bae et al. (2020) showed that asperphenin-A derived from *Aspergillus* sp. led to significant antitumor activity and stress-induced apoptosis in the colon cancer cell line [74]. Prabhu et al. (2014) recorded that *Aspergillus japonicas* Saito showed stress-induced and caspase-dependent anticancer potential against the cervical cancer (HeLa) cell line [75]. Ghfar et al. (2021) observed that *Aspergillus terreus*-derived terretonin N and butyrolactone I exhibited antitumor efficacy, with an IC_50_ value of 7.4 µg/mL against metastatic prostate cells (PC-3), and with an IC_50_ value of 1.2 µg/mL against ovarian adenocarcinoma cells [76]. In the present study, programmed cell death was initiated by releasing cytochrome C from the mitochondrial membrane into the cytosol, which binds to the apoptotic precursor (procaspase 9) proteins that facilitate early apoptosis. These results suggest that the bioactive compounds from these particular fungal strains can be used commercially to produce novel antibiotics and anticancer drugs after purification and proper standardization. 

## 5. Conclusions

The present study confirms the biological activities of the rhizospheric-derived *Aspergillus niger* strain AK-6. The in vitro investigations of ethyl acetate extracts of the *Aspergillus niger* strain AK-6 lead us to infer its possible antimicrobial activity against human Gram-negative and Gram-positive bacteria, as well as yeast pathogens. We also noted a promising antifungal potential against phytopathogens. Furthermore, the FTIR analysis revealed the presence of different functional groups, and the GC-MS analysis hinted at the presence of bioactive metabolites known for various pharmaceutical applications. Further, the cytotoxicity of the ethyl acetate extract of the *Aspergillus niger* strain AK-6 against the MCF-7 cancer cell line (human breast adenocarcinoma) showed dose-dependent antiproliferative efficacy, inducing cell death and triggering initial and late apoptosis in the tested cancer cell line. The study’s outcomes imply future research directions, aiming at the in vivo study and determination of the mechanisms of action. Overall, the study concludes that the isolated *Aspergillus niger* strain AK-6 has great potential for use in the discovery of new antimicrobial, antifungal and anticancer drugs, and can be used as a therapeutic model in the pharmaceutical and agricultural sectors. 

## Figures and Tables

**Table 1:** Tukey’s multiple comparison tests between groups. *p* < 0.05 (*), *p* < 0.01 (**), *p* < 0.001 (***), and *p* < 0.0001 (****).

Tukey’s Multiple Comparison Test	Mean Diff.	95.00% CI of Diff.	Below Threshold?	Summary	Adjusted *p* Value
25 µL					
*C. albicans* vs. *E. coli*	1.423	0.4702 to 2.376	Yes	***	0.0006
*C. albicans* vs. *B. subtilis*	1.217	0.2636 to 2.170	Yes	**	0.005
*C. albicans* vs. *S. aureus*	14.33	13.38 to 15.29	Yes	****	<0.0001
*E. coli* vs. *S. flexneri*	−2.05	−3.003 to −1.097	Yes	****	<0.0001
*E. coli* vs. *S. aureus*	12.91	11.96 to 13.86	Yes	****	<0.0001
*S. flexneri* vs. *B. subtilis*	1.843	0.8902 to 2.796	Yes	****	<0.0001
*S. flexneri* vs. *S. aureus*	14.96	14.01 to 15.91	Yes	****	<0.0001
*B. subtilis* vs. *S. aureus*	13.12	12.16 to 14.07	Yes	****	<0.0001
50 µL					
*K. pneumoniae* vs. *S. flexneri*	−3.037	−3.990 to −2.084	Yes	****	<0.0001
*C. albicans* vs. *S. flexneri*	1.12	0.1669 to 2.073	Yes	*	0.0123
*C. albicans* vs. *B. subtilis*	2.063	1.110 to 3.016	Yes	****	<0.0001
*E. coli* vs. *B. subtilis*	1.73	0.7769 to 2.683	Yes	****	<0.0001
*E. coli* vs. *S. aureus*	6.84	5.887 to 7.793	Yes	****	<0.0001
*S. flexneri* vs. *S. aureus*	6.053	5.100 to 7.006	Yes	****	<0.0001
*B. subtilis* vs. *S. aureus*	5.11	4.157 to 6.063	Yes	****	<0.0001
75 µL					
*K. pneumoniae* vs. *S. flexneri*	−3.27	−4.223 to −2.317	Yes	****	<0.0001
*K. pneumoniae* vs. *S. aureus*	1.03	0.07689 to 1.983	Yes	*	0.0268
*C. albicans* vs. *S. aureus*	4.933	3.980 to 5.886	Yes	****	<0.0001
*E. coli* vs. *S. aureus*	4.86	3.907 to 5.813	Yes	****	<0.0001
*S. flexneri* vs. *S. aureus*	4.3	3.347 to 5.253	Yes	****	<0.0001
*B. subtilis* vs. *S. aureus*	4.27	3.317 to 5.223	Yes	****	<0.0001
100 µL					
*K. pneumoniae* vs. *E. coli*	−3.017	−3.970 to −2.064	Yes	****	<0.0001
*K. pneumoniae* vs. *S. flexneri*	−2.657	−3.610 to −1.704	Yes	****	<0.0001
*C. albicans* vs. *E. coli*	1.043	0.09022 to 1.996	Yes	*	0.024
*C. albicans* vs. *S. aureus*	3.157	2.204 to 4.110	Yes	****	<0.0001
*E. coli* vs. *B. subtilis*	−1.187	−2.140 to −0.2336	Yes	**	0.0067
*S. flexneri* vs. *B. subtilis*	−1.547	−2.500 to −0.5936	Yes	***	0.0002

**Table 2:** FTIR analysis of ethyl acetate extract of *Aspergillus niger* strain AK-6 showing different functional groups.

Absorption	Peak Appearance	Group	Compound Class
3437.24	Medium	O–H stretching	Alcohol
2923.96	Sharp	C–H stretching	Alkane
2852.40	Sharp	C–H stretching	Alkane
1612.04	Strong bond	C=C stretching	α, β-Unsaturated ketone
1497.19	Weak	N–O stretching	Nitro compound
1458.47	Medium	C–H bending	Alkane
1384.27	Strong	S=O stretching	Sulfate
1251.92	Medium	C–N stretching	Alkyl aryl ether
1120.97	Weak	C–O stretching	Aliphatic ether
549.67	Medium	C–I stretching	Halo compound

**Table 3:** Compounds present in ethyl acetate extract of *Aspergillus niger* strain AK-6 analyzed by GC-MS.

Peak	Retention Time	Peak Area %	Compound Name	Molecular Formula	Molecular Weight
1	11.069	7.02	1-Dodecene	C_10_H_21_CH=CH_2_	168.32
2	13.398	2.13	Tetradecane	C_14_H_3_O	198.39
3	16.344	1.67	Cyclodecane	(CH_2_)_12_	168.32
4	19.517	8.60	2,4-Ditert-butylphenol	C_14_H_22_O	204.32
5	25.615	7.77	E-14-Hexadecenal	C_16_H_30_O	3238.41
6	27.340	23.82	N-didehydrohexacarboxyl-2,4,5-trimethylpiperazine	C_13_H_22_N_2_O	222
7	29.207	14.65	Dibutyl phthalate	C_16_H_22_O_4_	278.34
8	29.602	8.98	E-15-Heptadecenal	C_17_H_32_O	252.43
9	33.252	6.01	1-Heneicosanol	C_2_H_44_O	312.58
10	36.614	2.35	Trifluroacetoxy hexadecane	C_18_H_33_F_3_O_2_	338.4
11	37.995	3.05	Cyclopentane, heneicosyl	C_26_H_52_	364.7
12	43.141	6.16	Squalene	C_30_H_50_	410.7
13	44.014	2.89	Tricosane	C_23_H_48_	324.63
14	45.467	2.74	Nonadecane	C_19_H_40_	268.518
15	47.184	2.16	Octadecane, 2 methyl	C_19_H_40_	268.52

**Table 4:** Table showing the percentage of cells that underwent apoptosis and necrosis in untreated, standard control- (std) and test compound (*Aspergillus niger* strain AK6)-treated MCF-7 cells in comparison to viable cells.

Cell Condition	Necrotic Cells (UL)	Late Apoptosis (UR)	Viable Cells (LL)	Early Apoptosis (LR)
Untreated	0	0	100	0
Positive control	3.97%	21.33	42.77	31.93
AK6	3.16	26.43	53.11	17.3

## Data Availability

The raw data used and/or analyzed during the current study will be available from the corresponding author on reasonable request.
